# Time to Expect More From Pharmacometrics

**DOI:** 10.1002/cpt.1914

**Published:** 2020-06-19

**Authors:** Jeffrey S. Barrett

**Affiliations:** ^1^ Bill & Melinda Gates Medical Research Institute Cambridge Massachusetts USA; ^2^ Present address: Critical Path Institute Tuscon Arizona USA

Pharmacometrics (PMX) has matured over the past 4 decades and become an integral part of drug development. Whereas the industrialization^1^ of the discipline continues to standardize and refine the deliverables, particularly those that become incorporated into regulatory submissions, PMX should still evolve further to embrace newer and complimentary methodologies and approaches as other quantitative sciences have. There are obvious adaptions that the discipline should embrace in order to achieve its potential.

## INDUSTRIALIZATION AT THE EXPENSE OF INNOVATION

The strong shadow cast by regulatory impact and drug development customers has so influenced the direction of the field that it has not fully embraced the technologic gains made by related disciplines. PMX has also not expanded its scope to develop more meaningful synergies with new disciplines in the quantitative sciences to further access the pool of potential candidates or participate in collaborative science outside of drug development. The impact of regulatory success has been a mixed blessing. On one hand, the field has demonstrated the value of modeling and simulation for internal decision making and confidence in regulatory decisions informed by such efforts consistent with the promise of model informed drug development.^2^, ^3^ On the other hand, the models used for such purposes remain in the graveyard of submission materials confined to the vault of regulatory documentation and seldom touch patients in any meaningful way. This situation has created an environment where the most visible deliverables for the discipline are analysis reports embedded in the submission and hopefully labeling references^3^, ^4^, ^5^, ^6^ that describe the impact of such analyses. Likewise, these deliverables have focused much of the PMX research toward the industrialization of the process and best practices.^1^

One of the more obvious ways to begin expanding the PMX utility within the existing support base is to derive new and improved milestones that better showcase the influence and also highlight new collaborative expansions to its utility for candidate selection (with system pharmacology, for instance), pediatric extrapolation (system pharmacology and artificial intelligence/machine learning (AI/ML) collaboration) and product differentiation (AI/ML integration with real‐world data analytics). Of course, having milestones outside of the drug development decision making process would be valuable as well, particularly in the areas of marketplace performance or personalized medicine. Opportunities to merge PMX with real‐world data and/or the development of drug dashboards to manage patient outcomes provide some evidence that this is possible.

## MODERN DATA SCIENCE AND PREDICTIVE ANALYTICS

**Figure 1:**
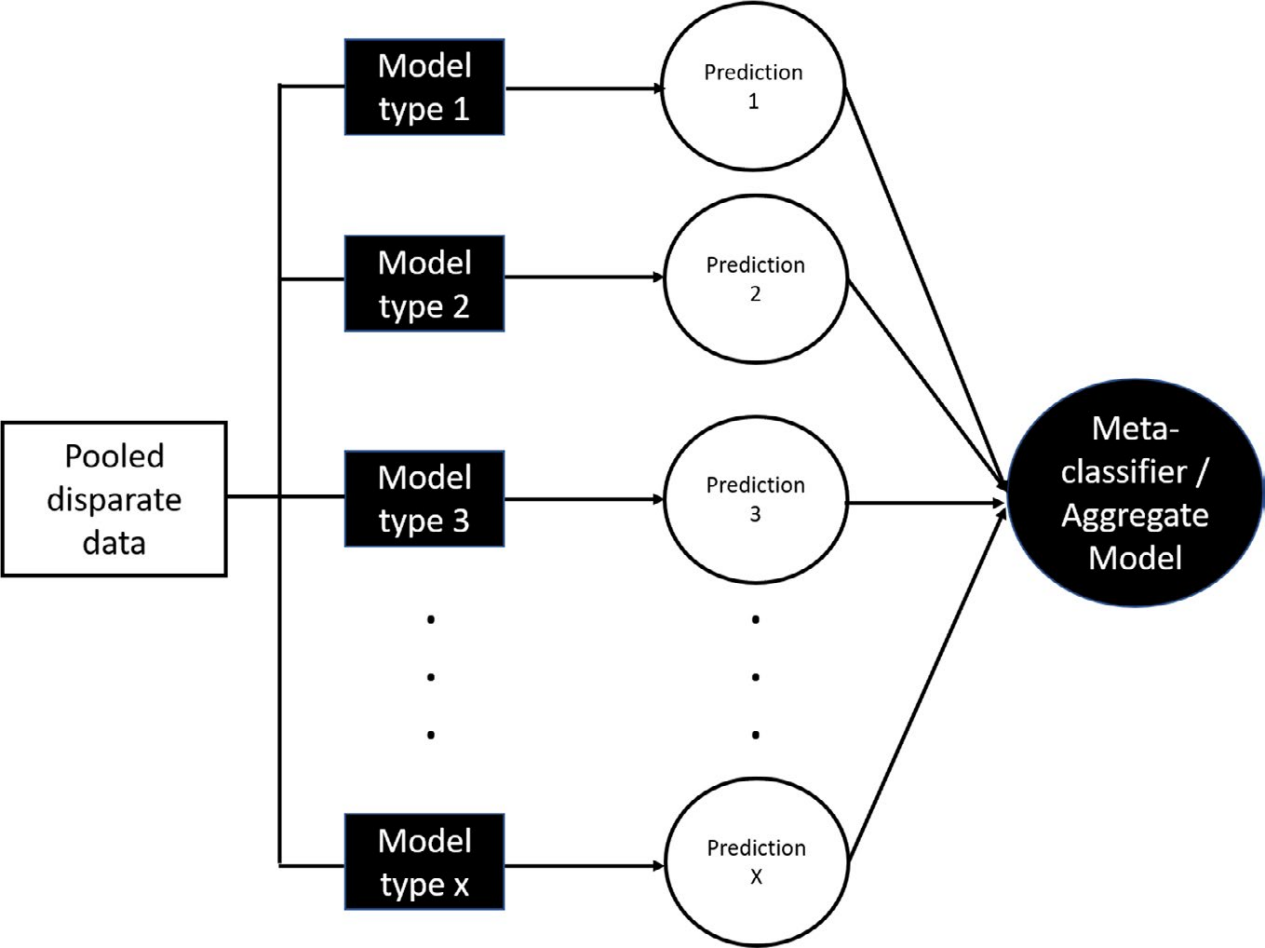
Ensemble model approach adapted for drug development.

There are too many smart people in our field to be satisfied with the status quo—a mental “hot flash” is needed to embrace the evolving data and quantitative sciences around us. The public recognition of AI and ML is evidence of the need to better promote the discipline beyond talking to Alexa or driving a Tesla. Better data to train predictive models in the life sciences would facilitate another important step for PMX; collaboration and sharing are essential in this regard. One of the more common approaches in predictive analytics is the utilization of ensemble models to solve problems. There is no magic to this really as it accommodates disparate data types and models that contribute information value to the entity being predicted. **Figure** **1** provides a schematic of the basic approach integrating disparate model types to predict outcomes of interest and using a weighting algorithm or utility function to project an aggregate prediction, which better reflects potential outcomes. The contributions of the simulated outcomes to the overall predicted response can, therefore, be influenced by non‐modeling, subject matter experts thus creating a more team‐based and multidisciplinary effort. This would also accommodate various models constructed from different data and avoid the tedious “bottom‐up” vs. “top‐down” debate while looking more holistically at contributions to predictions where confidence is highest. This should be appreciated as a more collaborative, integrated approach as opposed to simple model averaging, as it can create weighted input based on expert opinion independent of the modeling. A recent application of an ensemble approach to predict dengue transmission^7^ provides a good example of the approach and the assessment of predictive value. The dengue example illustrates how both disparate data types (climate and viral transmission data) can be incorporated into different model types (regression‐based and data‐centric, smoothing algorithms) to make predictions shown to be superior to the independent models. Several aspects of the example are, in fact, borrowed from meteorological approaches. Again, this is not model averaging as one would engage for the purpose of limiting/minimizing aberrant predictive behavior. Such an approach could be utilized for pediatric extrapolation where exposure‐response, population pharmacokinetic/pharmacodynamic and system pharmacology models could address disease similarity, target exposure, and dosing considerations in one model venue from very different perspectives and underlying structural models. There is consistent feedback from regulators on the value of complimentary approaches to enhance confidence in predictions but having these approaches combined to more effectively pressure test outcome scenarios would seemingly be viewed positively as well.

Predictive analytics has evolved and continues to do so by evaluating the quality of predictive performance as a metric. During industrialized PMX analyses, much retrodiction is completed under the guise of validation but very little prediction is actually evaluated beyond simulations to influence designs. A more formal report card on prospective prediction certainly must be a part of the discipline’s valuation in the future. Although some companies and the US Food and Drug Administration (FDA) have published on PMX influence on submissions and even decision making impact, there is little to describe quantitatively the “quality” of predictions. Specifically, how accurate were the predictions relative to the actual outcomes at the conclusion of the study? How good did they need to be? How good should a future model be for it to be more useful or clinically relevant? As we all appreciate that models evolve over time, such an approach would serve as an important baseline for consideration of model quality and performance. The PMX community could use this as a forum for more open dialogue, especially when communicating with the lay community as well. In the midst of the coronavirus disease 2019 (COVID‐19) pandemic, it is clear that there remains a gap in understanding and misinformation is problematic.

## LEARNING FROM OTHER DISCIPLINES AND INDUSTRIES

There is always much talk about leveraging the knowledge from other quantitative disciplines with many analogies to the application of modeling and simulation (M&S) from other quantitative disciplines, particularly the aerospace industry where reliance on M&S is essential to product design and launch with limited field testing. However, there are many other disciplines engaged in extensive use of M&S as part of their core or expanding business where PMX has had little or no outreach. More importantly, the nature of these shared learnings would be greatly enhanced by cross‐training opportunities and meaningful collaboration, not to mention broader appreciation for PMX to the lay community. Fields, such as sports betting, meteorology, and load repayment, invest heavily in quantitative sciences and enjoy great recognition among the lay community because of greater familiarity of their end‐products.

One path forward for PMX would be to more heavily invest in the M&S approaches used in the loan repayment, weather prediction (meteorology), and sports betting settings to better appreciate the integration of disparate data sources and to develop reasonable metrics for prediction accuracy. For instance, modeling the climate has a long and storied past with milestones on both the foundational models used as the backbone of many of today’s current models, compute history requirement evolution from the first Electronic Numerical Integrator and Computer (ENIAC) implementation to modern cloud‐based high‐performance computing, and the data requirements for more modern data‐centric models informed by ML approaches.^8^ Much can be learned from this history but specifically how (1) competing models can co‐exist and bring utility to outcome prediction (e.g., the European Center for Medium‐Range Weather Forecast (ECMWF) model and the National Weather Service’s Global Forecast System (GFS) model), and (2) how meteorologists are increasingly relying on ensemble systems to make predictions. Current implementation of this approach uses various simulations of the ensemble system to develop a family of alternative predictions by tweaking the initial conditions, capturing the range of uncertainty in forecasts. With this approach, forecasters gain a better understanding of the range of possible outcomes. An important learning from meteorology is that these models (European and American, for example) can coexist and be used for certain predictions under specific conditions. There is comfort in knowing the conditions under which one performs better than the other but no necessity for a universal model when both have utility.

Sports betting likewise provides an opportunity to learn new approaches and methodologies focused on improving prediction and the underlying simulation science has evolved to accommodate more complex nuances. Several good examples with predicting outcomes from National Basketball Association (NBA) games exist that illustrate how the field moved from win‐loss predictions to more fully describing the progression of the game with a microsimulation approach that utilizes possession‐based Markov model to project the entire game.^9^ Most importantly, these scientists have merged their field with advance analytics to better communicate their output in layman’s terms. This approach would seemingly be complementary to agent‐based clinical trial simulation models that try to describe population‐based infectious disease progression with heuristics describing seasonal and mosquito transmission aspects of the model. This coupled with binomial declaration (similar to win/loss functionality) for infection or disease prevention could also serve as an example of PMX translation for clinical trial simulation.

To facilitate the expansion of PMX into other disciplines, some version of intelligent swarming might be appropriate.^10^ As the goal of intelligent swarming is the effective outsourcing or insourcing of new, complex problems with the intention of aligning the best resource (or resources) to resolve an issue, swarming could promote such collaboration faster with more creative resolutions. It will be important that such an approach avoids the rigid hierarchy and arbitrary boundaries that exist in most corporate environments as well as professional societies and promotes a social hierarchy of both service and support.

## Funding

No funding was received for this work.

## Conflict of Interest

The author declared no competing interests for this work.
